# Effect of Thermocycling, Teeth, and Polymerization Methods on Bond Strength Teeth-Denture Base

**DOI:** 10.1155/2018/2374327

**Published:** 2018-06-04

**Authors:** Sandra Lúcia Andrade de Freitas, William Cunha Brandt, Milton Edson Miranda, Rafael Pino Vitti

**Affiliations:** ^1^Department of Prosthodontics, School of Dentistry, São Leopoldo Mandic, Campinas, SP, Brazil; ^2^Department of Implantology, School of Dentistry, University of Santo Amaro, São Paulo, SP, Brazil; ^3^Department of Prosthodontics, School of Dentistry, University of Taubaté, Taubaté, SP, Brazil

## Abstract

**Objective:**

To evaluate the shear bond strength between different artificial teeth and denture base polymerized by two polymerization methods submitted to thermocycling.

**Materials and Methods:**

Two acrylic resins were selected according to the polymerization method (water-bath and microwave), and four different artificial teeth (Biotone, Dentsply; Trilux, Vipi Dent; Premium 8, Heraeus Kulzer; Soluut PX, Yamahachi) were also tested. The polymerization of the acrylic resin was performed by using conventional cycle (8 h at 74°C) in water-bath and using two cycles (20 min at 270 W + 5 min at 360 W) by the microwave method. The shear bond strength was evaluated after 24 h of water storage at 37°C (immediately) and after the thermocycling test (5,000 cycles, 5–55°C). The shear bond strength (*n*=10) was performed using a universal testing machine (Instron 4411) at a crosshead speed of 1.0 mm/min. Modes of failures were classified as cohesive and adhesive. The data (MPa) were statistically analyzed by three-way ANOVA, and the mean values were compared by the Tukey test (*α* = 0.05).

**Results:**

In general, the polymerization by microwave showed the highest shear bond strength values, and Trilux artificial teeth had the lowest bond strength values (*p* < 0.05). Thermocycling did not affect the shear bond strength (*p* < 0.05). There was a predominance of cohesive failures for all groups.

**Conclusions:**

The chemical composition of the artificial teeth affects the bond strength, and the microwave method is preferable to perform the acrylic resin polymerization.

## 1. Introduction

The acrylic resin replaced the vulcanite used for manufacturing of denture bases [1]. The industrial plastics have performed modifications in order to improve the physicochemical, mechanical, and biological properties of the acrylic resins, such as increasing the tensile strength, providing excellent esthetics, maintaining the dimensional stability at different mouth temperatures, low solubility, and ease of processing and repair [2, 3]. In total or partial prosthetic rehabilitation, the use of acrylic resins for the preparation of denture bases has been shown over the years to be a reliable method with high survival rates documented in the literature [2, 4, 5].

The acrylic resin is available in two main forms as regards to the material polymerization method: heat-cured acrylic resin using hot water or microwave energy and self-cured acrylic resin. During the polymerization, occurs the conversion of the monomer (methyl methacrylate (MMA)) into polymer (polymethyl methacrylate (PMMA)) [2, 6]. However, this conversion is not complete, which results in an amount of residual monomer, which can directly affect the structural integrity of the polymer compromising its flexural strength, hardness, and biocompatibility [2, 7].

There are two cycles of polymerization (conventional and short) of heat-cured acrylic resin in hot water-bath. In the conventional cycle, the mode of activation is by immersing the flasks in water at 74°C for eight hours or more. The short cycle consists in immersing the flasks at 74°C for two hours and raising it to 100°C for one hour. However, in this cycle, the boiling temperature (100°C) must be carefully controlled avoiding porosities in the tooth-acrylic resin interface [2]. The acrylic resins polymerized by microwave energy demonstrate advantages such as greater technical convenience [8] as well as less distortion compared to water-bath polymerization [9].

Another issue to be considered is the shear tension that occurs in the artificial teeth bound to denture base (acrylic resin). The displacement of the artificial teeth occurs because the stresses at the tooth-resin interface exceed the bond strength between these materials [10]. The teeth movement in the prosthesis is a common situation in clinical practices where it is necessary to perform repairs on the prosthesis [11, 12]. However, this behavior is not yet fully understood, since it can be attributed to other factors including the direction of masticatory forces, contamination of the artificial teeth with wax, type of polymerization of heat-cured acrylic resin, and also chemical treatment of the cervical region of the acrylic resin teeth [13].

The main component present in the polymer matrix of acrylic resin teeth is PMMA. An acrylic resin that presents a polymer chain with a high cross-linking significantly reduces the adhesion between teeth-acrylic resin [1, 2, 12]. Thus, the choice of the type of the artificial tooth and the polymerization method of the acrylic resin to be used in the manufacture of the total dentures is important in order to promote adequate tooth-acrylic resin denture base bond strength.

The aim of this study was to evaluate the influence of the thermocycling on bond strength between different artificial teeth and acrylic resin polymerized by the conventional and microwave energy methods.

## 2. Materials and Methods

One hundred sixty first lower right molars (46) were used to evaluate the bond strength. Initially, two groups were created according to the polymerization method of the acrylic resin: heat-cured acrylic resin (Clássico, Clássico Artigos Odontológicos, São Paulo, SP, Brazil) polymerized by the conventional cycle (water-bath) and heat-cured acrylic resin (Onda-Cryl, Clássico Artigos Odontológicos) polymerized by the microwave energy. Each one of these groups was subdivided into four groups as per the type of artificial tooth used (Table 1).

The artificial teeth were inserted into a silicone matrix (Silicone Master, Talmax, Curitiba, PR, Brazil) with 15 mm high × 22 mm diameter. Thus, the height of the exposed tooth surface did not influence the values of the bond strength [14].

### 2.1. Inclusion of Artificial Teeth and Polymerization of the Heat-Cured Acrylic Resin

The assembly wax/artificial tooth was taken in a specific flask for each polymerization method: a plastic flask for the microwave method (Vipi Dent, Pirassununga, SP, Brazil) and a metal flask for the conventional method (OGP, Bragança Paulista, SP, Brazil). The type II dental stone (Durastone, Vitória, ES, Brazil) was used following the manufacturer's instructions (30 ml/100 g; water/powder ratio). The dental stone was mixed under vacuum (Renfert, Ribeirão Preto, SP, Brazil).

The flask was filled with dental stone and placed in a hydraulic press (1000 kgf) for 45 min. Then the wax was removed using water (100°C) for 10 min. All samples were washed with the anionic detergent (Limpol, Bombril, São Bernardo do Campo, SP, Brazil) and water, being inspected visually to ensure complete removal of the wax.

The heat-cured acrylic resin was prepared according to the manufacturer's instructions. The flask was subjected to hydraulic pressing. The metal flasks (conventional) were submitted to 1500 kgf and the plastic flasks (microwave energy) to 1000 kgf. Both flasks remained under pressure for 60 minutes. Microwave energy polymerization was performed by a cycle of 20 min at 270 W + 5 min at 360 W. The conventional polymerization method consisted of heating the acrylic resin at 74°C for 8 h. After the polymerization process, the flasks were placed on the bench at the room temperature for cooling. The samples were stored in distilled water at 37°C for 24 h (no thermocycling group).

### 2.2. Thermocycling

Half of the samples of each group were stored in distilled water (37°C). Thermocycling (MSCT-3, São Carlos, SP, Brazil) with 5,000 cycles between 5° and 55°C (dwell time of 30 s) was performed. This procedure corresponds to a five-year period of oral temperature conditions [15, 16].

### 2.3. Shear Bond Strength Test

All samples (*n*=10) were subjected to the shear test using a universal mechanical testing machine (Instron 4411, Instron Inc., Canton, MA, USA). The samples were fixed in a PVC cylinder perpendicular to the force applied. A 5000 N load cell was used with a crosshead speed of 1.0 mm/min until fracture of acrylic tooth-resin. The bond strength data were obtained in kN and converted to MPa. The fracture modes of the samples were analyzed visually and classified as adhesive (between artificial tooth and acrylic resin) or cohesive (artificial tooth or acrylic resin) failures [17].

### 2.4. Statistical Analysis

The data were statistically analyzed by the Kolmogorov–Smirnov nonparametric test. The data were submitted to analysis of variance (3-way ANOVA), and the mean values were compared by the Tukey test (*α* = 0.05).

## 3. Results

The statistical analysis showed no significant effect for the interaction among all three study factors (*p*=0.522); for interactions between artificial teeth and polymerization methods (*p*=0.137), artificial teeth and storage methods (*p*=0.096), and polymerization and storage methods (*p*=0.408); and for storage methods (*p*=0.153). However, significant difference was found for artificial teeth (*p* < 0.05) and polymerization methods (*p* < 0.05).

Table 2 shows that the microwave energy polymerization method had the highest bond strength values for all teeth (*p* < 0.05), except for the Biotone, which presented statistically similar mean values between the polymerization methods (*p* > 0.05).

In general, Trilux presented the lowest values of bond strength (*p* < 0.05), except for the group without thermocycling with the microwave polymerization method, where all the teeth presented similar results and for the group with thermocycling and microwave energy, where Trilux was statistically similar to the Biotone and Soluut teeth (*p* > 0.05).

There was a predominance of cohesive failures for all groups accordingly (Table 3).

## 4. Discussion

In this study, the evaluation of the acrylic resins as a function of the polymerization methods, independent of the tooth, showed that the acrylic resin polymerized by microwave energy had higher bond strength values when compared to polymerization in water-bath, rejecting the null hypothesis. These results are in agreement with other studies, since the acrylic resin polymerized by microwave energy presented lower porosity, better clinical performance, and mechanical properties, besides higher bond strength values than water-bath [4, 9, 10, 18].

The energy emitted by the microwave oven allows the vibration of water molecules two to three billion times per second. This agitation produces friction between water molecules resulting in water heating [19]. In the same way as water, the monomer molecules present in the acrylic resin are agitated by the electromagnetic wave generated, and the friction of these molecules promotes the release of the heat necessary for the conversion of the monomer into polymer [20]. Polymerization by microwave energy has three advantages: decrease of polymerization time, prevention of internal porosities in the resins, and increase in the degree of conversion [9, 20, 21]. These advantages are derived from the dielectric heat that provides an immediate, rapid, and uniform heating of the entire acrylic resin, providing a smooth and polished surface on the prosthesis, avoiding the accumulation of biofilm, and less discoloration of the prosthesis [17].

The microwave energy can be used to complement the polymerization reaction of the heat-cured acrylic resin in order to decrease the residual monomer [20]. The results show that the conventional polymerization method presented the lowest bond strength values. This result is due to the porosities present at the tooth-acrylic resin interface and/or due to the high levels of residual monomer: once spaces are caused by the porosity, they may promote the degradation of this interface. Furthermore, the residual monomer generates a plasticizing effect, reducing the mechanical properties of the acrylic resins and adhesion with the artificial teeth [13].

This study also evaluated the effect of thermocycling on the bond strength between artificial tooth and acrylic resin denture base. The results demonstrated that the thermocycling did not affect the bond strength in both polymerization methods and all teeth tested. Some studies have shown that the thermocycling has a deleterious effect on the bond strength between artificial tooth and acrylic resin, since the thermocycling causes hydrolytic degradation and consequent fracture or displacement of the teeth [22, 23]. However, in the present study independent of the polymerization method and the tooth type, the thermocycling did not affect the bond strength, corroborating with other studies [15, 24]. The differences between the results can be related to the number of cycles used in each study (cycles above 10,000), besides the time and the storage temperature of the samples [22].

The results of this study show differences on the bond strength between the different teeth and the acrylic resin. Trilux artificial teeth obtained the lowest bond strength values in both polymerization methods. A possible explanation for this behavior could be related to the composition of the artificial teeth. Artificial teeth are primarily composed of PMMA and fillers. Trilux is made by a double cross-link (DLC) composed of PMMA, EDMA, and fillers [25, 26]. Other artificial teeth have the association of PMMA with the addition of cross-linking agent resins with interpenetrating polymer network (IPN) improving their bond strength and fracture resistance [27]. IPN is formed by a polymer chain crossed inside another chain occupied with a second polymer, and a polymer chain with a high degree of cross-linking cannot be separated without chemical bond rupture. This factor may produce artificial teeth with better mechanical and bond strength properties [28].

Furthermore, the components of the Trilux may increase the amount of free polymer chains in the artificial tooth, decreasing its physicochemical properties. This increase of the free polymers affects the bond strength of these teeth with monomer of the heat-cured acrylic resin used for denture base [25]. A previous study showed that Trilux are composed essentially of carbon, oxygen, hydrogen (main elements), and silicon, but it has more silicon and carbon than other artificial teeth such as Biotone and Soluut [29].

However, some studies did not find any connection between the chemical composition (cross-linking agents × conventional PMMA) and the bond strength of the artificial teeth. These conflicting results could be explained by experimental design differences (measuring devices and bond strength methods). Moreover, the most failures (more than 80%) were cohesive in the acrylic resin and/or artificial teeth. This result reflects the resistance of the artificial tooth or acrylic resin [17]. In either case, for most of the samples, a predominantly cohesive failure may suggest that the bond strength between the tooth and acrylic resin was greater than the resistance of either material alone.

The results are of clinical relevance, once the different cycles of polymerization and artificial teeth affect the bond strength. But, clinically, the bond strength is also influenced by other factors such as mastication pattern, antagonists, type of denture, dental attrition, type of toothbrush, and products used to clean the prosthesis. Further studies are needed to elucidate if the differences found between the properties of artificial teeth in the laboratory have any effect on their clinical behavior. It emphasized that these variables deserve to be studied since there is a growing concern of dental surgeons with the stability of prostheses, always aiming for the best clinical prognosis of rehabilitative prosthodontic treatment.

## 5. Conclusion

The microwave energy promoted the highest bond strength teeth-denture base value, and the chemical composition of the artificial tooth influences adhesion between tooth and denture base. Thermocycling did not affect the bond strength teeth-denture base.

## Figures and Tables

**Table 1:** Materials-brand name, manufacturers, and batch numbers.

Brand name	Manufacturer	Composition	Batch number
Biotone	Dentsply	Cross-linked PMMA (IPN)	077279
Premium 8	Heraeus Kulzer	MPM, prepolymerized PMMA (IPN)	1412032925
Trilux	Vipi Produtos Odontológicos	PMMA, EDMA (DCL)	1303948083
Soluut	Yamahachi Kota Imports	Methacrylate fluoride	305931

PMMA: polymethyl methacrylate; IPN: interpenetrating polymer networks; MPM: multiplex polymer matrix; EDMA: ethylene glycol dimethacrylate; DCL: double cross-link.

**Table 2:** Mean values (±SD) of shear bond strength (MPa) of the artificial teeth and polymerization methods with or without thermocycling.

Teeth	Without thermocycling		With thermocycling	
	Conventional	Microwave	Conventional	Microwave
Premium	4.12 (1.08) A,b	6.68 (1.17) A,a	3.90 (0.76) A,b	7.24 (1.80) A,a
Trilux	2.74 (0.63) B,b	5.61 (0.79) A,a	1.78 (0.40) B,b	4.46 (1.44) B,a
Soluut	4.00 (1.12) A,b	6.00 (1.13) A,a	3.82 (0.77) A,b	5.62 (0.47) AB,a
Biotone	4.45 (1.16) A,a	5.78 (0.84) A,a	4.11 (1.14) A,a	5.65 (132) AB,a
Pool mean	4.92 (1.59)^*∗*^		4.57 (1,89)^*∗*^	

Different letters indicate statistically significant difference (*p* < 0.05): capital letters for comparison between artificial teeth (columns) and small letters for comparison between polymerization methods within each storage method (in rows). ^*∗*^Comparison between storage times.

**Table 3:** Modes of failures (%).

	Premium 8	Trilux	Soluut	Biotone
Cohesive	82.5%	85%	82.5%	80%
Adhesive	17.5%	15%	17.5%	20%

## Data Availability

The data, mean values, and standard deviation of shear bond strength (MPa) as well as modes of failures data used to support the findings of this study are available from the corresponding author upon request.

## References

[1] Alla R. K., Swamy R., Vyas R. (2015). Conventional and contemporary polymers for the fabrication of denture prosthesis: part I-overview, composition and properties. *International Journal of Applied Dental Sciences*.

[2] Gettleman L., Phoenix R. D., Rawls H. R., Anusavice K. J., Shen C., Rawls H. R. (2013). Prosthetic polymers and resins. *Phillips’ Science of Dental Materials*.

[3] Agha H., Flinton R., Vaidyanathan T. (2016). Optimization of fracture resistance and stiffness of heat-polymerized high impact acrylic resin with localized e-glass FIBER FORCE® reinforcement at different stress points. *Journal of Prosthodontics*.

[4] Radford D. R., Juszczyk A. S., Clark R. K. (2014). The bond between acrylic resin denture teeth and the denture base: recommendations for best practice. *British Dental Journal*.

[5] Rashid H., Sheikh Z., Vohra F. (2015). Allergic effects of the residual monomer used in denture base acrylic resins. *European Journal of Dentistry*.

[6] Ozkomur A., Fortes C. B., Beatriz C. (2016). Effects of postpolymerization microwave irradiation on provisional dental acrylics: physical and mechanical properties. *Journal of Applied Biomaterials and Functional Materials*.

[7] Fonseca R. B., Kasuya A. V., Favarão I. N. (2015). The influence of polymerization type and reinforcement method on flexural strength of acrylic resin. *Scientific World Journal*.

[8] Savirmath A., Mishra V. (2016). A comparative evaluation of the linear dimensional changes of two different commercially available heat cure acrylic resins during three different cooling regimens. *Journal of Clinical and Diagnostic Research*.

[9] Singh S., Palaskar J. N., Mittal S. (2013). Comparative evaluation of surface porosities in conventional heat polymerized acrylic resin cured by water bath and microwave energy with microwavable acrylic resin cured by microwave energy. *Contemporary Clinical Dentistry*.

[10] Silva-Concílio L. R., Meloto C. B., Neves A. C. (2012). Influence of different flasking and polymerizing methods on the occlusal vertical dimension of complete dentures. *Acta Odontologica Latinoamericana*.

[11] Negreiros W. A., Consani R. L. X., Mesquita M. F. (2009). Effect of flask closure method and post-pressing time on the displacement of maxillary denture teeth. *Open Dentistry Journal*.

[12] Lopes M. C., Consani R. L. X., Mesquita M. F. (2011). Effect of monomer content in the monomer-polymer ratio on complete denture teeth displacement. *Brazilian Dental Journal*.

[13] Barbosa D. B., de Souza R. F., Pero A. C., Marra J., Compagnoni M. A. (2007). Flexural strength of acrylic resins polymerized by different cycles. *Journal of Applied Oral Science*.

[14] Mahadevan V., Krishnan M., Krishnan C. S., Azhagarasan N. S., Sampathkumar J., Ramasubramanian H. (2015). Influence of surface modifications of acrylic resin teeth on shear bond strength with denture base resin-an in vitro study. *Journal of Clinical and Diagnostic Research*.

[15] Chaves C. A. L., Regis R. R., Machado A. L., de Souza R. F. (2009). Effect of ridge lap surface treatment and thermocycling on microtensile bond strength of acrylic teeth to denture base resins. *Brazilian Dental Journal*.

[16] Schneider R. L., Curtis E. R., Clancy J. M. S. (2002). Tensile bond strength of acrylic resin denture teeth to a microwave-or heat-processed denture base. *Journal of Prosthetic Dentistry*.

[17] Yadav N. S., Somkuwar S., Mishra S. K. (2015). Evaluation of bond strength of acrylic teeth to denture base using different polymerization techniques: a comparative study. *Journal of International Oral Health*.

[18] Arioli-Filho J. N., Butignon L. E., Pereira R. P., Lucas M. G., de Assis Mollo Junior F. (2001). Flexural strength of acrylic resin repairs processed by different methods: water bath, microwave energy and chemical polymerization. *Journal of Applied Oral Science*.

[19] Simon C. J., Dupuy D. E., Mayo-Smith W. W. (2005). Microwave ablation: principles and applications. *Radiographics*.

[20] Paes-Junior T. J. A., Carvalho R. F., Cavalcanti S. C. M., de Siqueira Ferreira Anza Saavedra G., Borges A. L. S. (2013). Influence of plaster drying on the amount of residual monomer in heat-cured acrylic resins. *Brazilian Journal of Oral Sciences*.

[21] Kasina S. P., Ajaz T., Attili S. (2014). To evaluate and compare the porosities in the acrylic mandibular denture bases processed by two different polymerization techniques, using two different brands of commercially available denture base resins—an in vitro study. *Journal of International Oral Health*.

[22] Saavedra G., Valandro L. F., Leite F. P. P. (2007). Bond strength of acrylic teeth to denture base resin after various surface conditioning methods before and after thermocycling. *International Journal of Prosthodontics*.

[23] Marra J. S., Freitas R., Barbosa D. B., Pero A. C., Compagnoni M. A. (2009). Evaluation of the bond strength of denture base resins to acrylic resin teeth: effect of thermocycling. *Journal of Prosthodontics*.

[24] Barbosa D. B., Barão V. A., Monteiro D. R., Compagnoni M. A., Marra J. (2008). Bond strength of denture teeth to acrylic resin: effect of thermocycling and polymerisation methods. *Gerodontology*.

[25] Korkmaz T., Dogan A., Dogan O. M. (2011). The bond strength of a highly cross-linked denture tooth to denture base polymers: a comparative study. *Journal of Adhesive Dentistry*.

[26] Hao Z., Yin H., Wang L., Meng Y. (2014). Wear behavior of seven artificial resin teeth assessed with three-dimensional measurements. *Journal of Prosthodontics*.

[27] Suzuki T., Takahashi H., Arksornnukit M., Oda N., Hirano S. (2005). Bonding properties of heat-polymerized denture base resin to Ti-6Al-7Nb alloy. *Dental Materials Journal*.

[28] Suzuki S. (2004). In vitro wear of nano-composite denture teeth. *Journal of Prosthodontics*.

[29] Grando M., Pacheco L. M., Botega D. M., Hirakata L. M., Balbinot Hilgert J. (2015). Artificial teeth: evaluation of wear resistance, microhardness and composition. *Revista Gaúcha de Odontologia*.

